# Therapeutic Efficacy of Vitamin E **δ**-Tocotrienol in Collagen-Induced Rat Model of Arthritis

**DOI:** 10.1155/2014/539540

**Published:** 2014-07-10

**Authors:** Nagaraja Haleagrahara, Mirashini Swaminathan, Srikumar Chakravarthi, Ammu Radhakrishnan

**Affiliations:** ^1^Discipline of Physiology and Pharmacology, School of Veterinary and Biomedical Sciences, Faculty of Medicine, Health and Molecular Sciences, James Cook University, Townsville QLD 4811, Australia; ^2^Department of Pathology, Faculty of Medicine, International Medical University, 57000 Kuala Lumpur, Malaysia; ^3^Perdana University Graduate School of Medicine, 57000 Kuala Lumpur, Malaysia

## Abstract

Rheumatoid arthritis (RA) is a chronic, systemic, inflammatory disease primarily involving inflammation of the joints. Although the management of the disease has advanced significantly in the past three decades, there is still no cure for RA. The aim of this study was to determine the therapeutic efficacy of *δ*-tocotrienol, in the rat model of collagen-induced arthritis (CIA). Arthritis was induced by intradermal injection of collagen type II emulsified in complete Freund's adjuvant. CIA rats were orally treated with *δ*-tocotrienol (10 mg/kg) or glucosamine hydrochloride (300 mg/kg) from day 25 to 50. Efficacy was assessed based on the ability to reduce paw edema, histopathological changes, suppression of collagen-specific T-cells, and a reduction in C-reactive protein (CRP) levels. It was established that *δ*-tocotrienol had the most significant impact in lowering paw edema when compared to glucosamine treatment. Paw edema changes correlated well with histopathological analysis where there was a significant reversal of changes in groups treated with *δ*-tocotrienol. The results suggest that *δ*-tocotrienol is efficient in amelioration of collagen-induced arthritis. Vitamin E delta-tocotrienol may be of therapeutic value against rheumatoid arthritis.

## 1. Introduction

Rheumatoid arthritis is a chronic inflammatory and destructive arthropathy. The worldwide occurrence of RA is about 1–1.5% of the population and it does not discriminate between age and ethnicity [1]. The precise aetiology of RA remains unknown. It has been established however that this disease is strongly linked with major histocompatibility complex (MHC) class II antigens, suggesting a genetic predisposition [2]. The pathogenesis of RA is associated with the activation of both cellular and humoral immune responses to an autoantigen [2, 3]. The activation of such responses leads to a potentiation of various proinflammatory cytokines (TNF-*α*, IL-1*β*, etc.), cascading into a vicious cycle of antigenic stimulation, inflammation, and joint destruction [4]. Currently, only symptomatic treatment of RA is available. Treatment for RA is unsatisfactory, as it consists of drugs with serious side effects and does not correct the underlying causes of arthritis. Therefore, there is a dire need for a safer and equally, if not more, efficient treatment option which is able to attack the root of the disease itself.

Nutraceuticals are loosely defined as “functional foods” and food products that provide medicinal or health benefits [5]. With a reported market value of US$ 75 billion, this industry is becoming progressively popular, partly due to the public's perception that “natural is better” [6]. Joining a long list of nutraceuticals are the tocotrienols, a constituent of vitamin E found in palm oil as a phytonutrient [7]. Vitamin E is a collective name for a complex mixture of homologues. The two main homologues of vitamin E are the tocopherols (T) and tocotrienols (T3). Each homology is subsequently composed of four isomers, *α*, *β*, *γ*, and *δ*. Tocomonoenol, found in small quantities in palm oil and marine organisms, forms the third homologue of vitamin E. Two isomers of tocomonoenol have been described to date with not much known about either [8]. Gaining substantial momentum in the past decade, tocotrienol research has led to the discovery of many of its important properties. Ranging from anticancer [9] to neuroprotective qualities [10], it is clear that tocotrienol use in various disease states is warranted. One such disease is rheumatoid arthritis (RA). The anti-inflammatory effects of tocotrienols have been less studied. Only recently, it has come to light that these isomers of vitamin E might bear some consequences on eradicating chronic inflammatory diseases such as arthritis, atherosclerosis, and coeliac disease to name a few. The aim of the present study is to assess the efficacy of *δ*-tocotrienol supplementation as a therapeutic agent in the collagen-induced rat model of arthritis.

## 2. Materials and Methods

Female Dark Agouti (DA) rats, 6–10 weeks old (150–200 g), were obtained from the Institute of Medical Research (IMR), Malaysia. Rats were maintained in individually ventilated cages (4 per cage) in the Animal Holding Facility (AHF) at the International Medical University (IMU) after their arrival. Food and water were available to the animals* ad libitum*. The AHF environment was climate-controlled with a 12-hour day and 12-hour night cycles. The International Medical University's joint committee for research and ethics approved all experimental procedures of this study. Delta-tocotrienol was a kind gift from Davos Life Sciences (Singapore). Collagen from chicken sternal cartilage type II, complete Freund's adjuvant (CFA), glucosamine hydrochloride, and acetic acid 99.8% were obtained from Sigma (Sigma Aldrich, USA).

Rats were divided randomly into four experimental groups (*n* = 6 in each group), control, arthritis alone, arthritis with *δ*-tocotrienol treated, and arthritis with glucosamine treated. Collagen from chicken sternal cartilage (5 mg) was reconstituted in 5 mL of 0.1 M cold acetic acid. The collagen was left to solubilise overnight at 4°C. Once the solution had become clear, complete Freund's adjuvant (CFA) at a ratio of 1 : 1 was added to the preparation of collagen. The mixture was then transferred to a handheld homogeniser and emulsified for approximately 20 minutes. For collagen-induced arthritis, the rats were briefly anesthetized and approximately 0.2–0.4 mL of the collagen-CFA emulsion was injected intradermally at the base of the tail at day 0. For the treatment group 10 mg/kg body weight of *δ*-tocotrienol was administered daily using feeding needles from day 25 to day 50. The amount administered was at a concentration of 10 mg/kg rat based on the weight of the rat at day 25. Treatment was administered as oral supplementation daily through oral gavage from day 25 to day 50. Arthritis with glucosamine group received 300 mg/kg body weight of glucosamine hydrochloride daily through oral gavage from day 25 to day 50.

The body weight of the animals was measured at five intervals. Rats were monitored daily for general appearance and behavior. The severity of arthritis was quantified by measuring the rat paw thickness using a digital vernier caliper at five-day intervals. Paw measurements were taken at each limb at four different joint positions. Twenty-four hours after the last day of the experiment, rats were anesthetized and blood sample was collected by cardiac puncture. Joint samples were also collected for histopathology. Spleen was removed and placed in a Petri dish containing approximately 5 mL RPMI media. Cells were immediately harvested and stored on ice. The plasma C-reactive protein (CRP) levels were quantified using the Millipore rat C-reactive protein ELISA kit.

### 2.1. Collagen Stimulation of Splenic Leukocytes

For collagen stimulation of splenic leukocytes, the tubes containing the cell suspension were centrifuged at 800 rpm for 5 minutes after which the supernatant was discarded. 1 mL of complete RPMI media and 3 mL of RBC lysis buffer were added to the pelleted cells. The tube was inverted gently several times for 30 seconds to ensure mixture of the buffer and the pellet. Then, the tubes were centrifuged again at 800 rpm for 5 minutes and the supernatant was discarded. The pellet was then resuspended in 2 mL of RPMI media. For the counting of cells, 100 *μ*L of the cell suspension is diluted with 900 *μ*L of complete RPMI medium. Then, 20 *μ*L of this suspension was transferred into a microcentrifuge tube and 20 *μ*L of trypan-blue dye was added to the cells. The trypan-blue dye stains dead cells and this will help to eliminate the counting of these cells. The suspension was transferred onto a glass haemocytometer and viewed under the microscope. Viable leukocytes were counted. The cell number was adjusted to 5 × 10^6^ cells/mL using the culture medium. About 200 *μ*L of this cell suspension was added in triplicate to the wells of a sterile 96-well flat-bottomed plate in the presence of 5 *μ*g/mL collagen. The plate was left to incubate for 72 hours in a humidified CO2 incubator at 37°C. Cell proliferation was determined using the MTT assay.

### 2.2. Histopathology

After being fixed in 10% formalin for one week, the joint tissues were transferred to a tube containing a decalcifying agent. Following decalcification, the tissue was processed using an automated tissue processor and embedding station. Blocks were sectioned at 3-4 *μ*m thickness and slides were prepared and stained (H&E).

### 2.3. Statistical Analysis

All data were analyzed using SPSS version 18 (SPSS Inc., Chicago, IL, USA). One-way analysis of variance (ANOVA) was used to detect differences among the experimental groups. For detecting differences between any two groups in a multiple group comparison, Tukey's test was used to evaluate paw oedema data and readings obtained from the ELISA. For all tests, a *P* value of less than 0.05 was considered to be significant.

## 3. Results

### 3.1. The Severity of Arthritis

**Figure 1:**
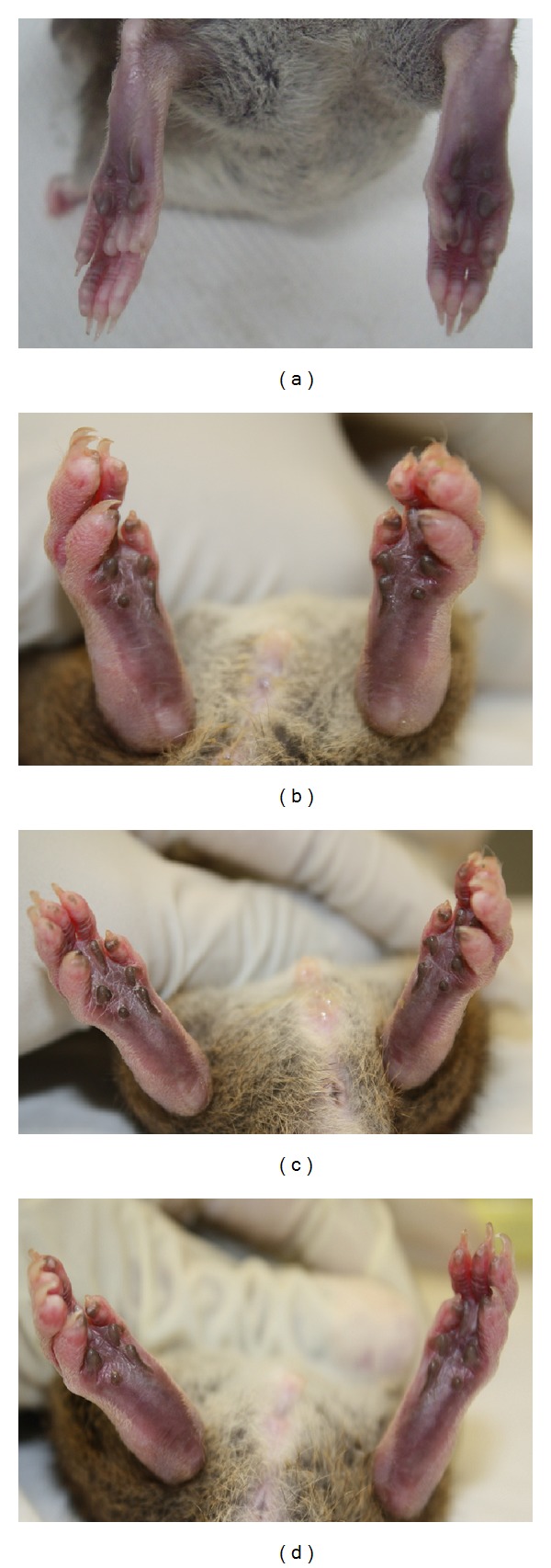
Effect of delta-tocotrienol on hind-paw edema. Hind-paws on day 50 of the experiment. Normal rats (a) from control group showed no signs of joint inflammation. Gross morphology of the hind-paws of arthritis rats showed maximal swelling and redness over joints (b). Treatment with tocotrienol and glucosamine reduced the severity of paw inflammation ((c) and (d)).

Arthritic animals began to have restrictions in movement at around day 20 where signs of arthritis began to develop. Some animals had developed a limp and moved around dragging their paws. Eating patterns, however, remained normal as denoted by a linear weight gain. As treatment progressed from day 25 to day 50, the mobility of the rats supplemented with either glucosamine or tocotrienol improved and almost all the rats in these groups regained ability to move around freely. Apart from the arthritis that affected the joints of these animals, there were no other gross changes observed (Figure 1).

### 3.2. Paw Edema

Paw edema was quantified by measuring paw size using a digital caliper from day 25 to 50. Arthritic animals began to show signs of arthritis between days 15 and 20. A total of 16 joints were measured for changes in swelling. Prior to induction, all rats showed no signs of arthritis or any paw deformities (Figure 1(a)). Signs of arthritis observed included swelling and redness over the joints, development of a limp, and tenderness to touch. In all rats, more than two joints were involved on each limb. As paw edema was most prominent on the hind-paws; only the joints in the hind-paws were assessed analytically using Tukey's post hoc test. Joints showed a significant (*P* < 0.05) decrease in paw edema for all treated groups when compared to the untreated group (Table 1 and Figures 1(c) and 1(d)). Comparison between tocotrienol and glucosamine revealed a significantly higher (*P* < 0.05) effect of *δ*-tocotrienol in reducing paw edema (Table 1). In the supplement-treated groups, swelling and redness over the joints reduced markedly and the rats regained their mobility. There was no recurrence of oedema in the joints and no additional joints became involved during this period (Table 1 and Figure 1).

### 3.3. Body Weight

**Figure 2:**
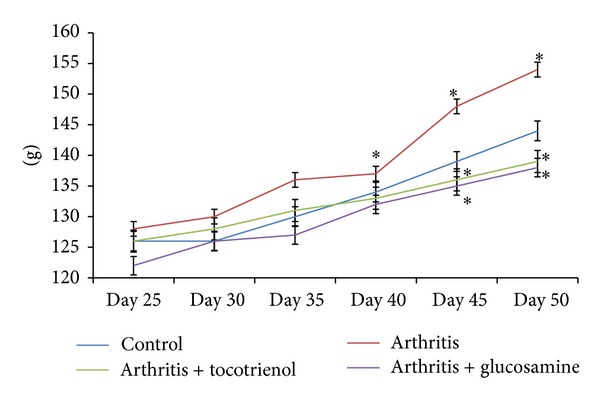
Effect of delta-tocotrienol on body weight changes in arthritis rats. Data are expressed as mean ± standard error. *P < 0.05: arthritis versus other groups.

Body weight of each rat was measured and recorded every five days from day 25 to 50. Although the starting weight for each group differed slightly, there was a noticeable upward trend for all groups (*P* < 0.05). The body weight of the animals ranged between 123.1 g and 138.9 g on day 35 and between 132.4 g and 156.8 g on day 50. The average body weight of all the animals rose between 10 and 12 g throughout the treatment period. The arthritis alone group had the highest ending weight. There was a significant increase in the body weight in this group after day 40 when compared with the other three groups (*P* < 0.05). The arthritic rats supplemented with *δ*-tocotrienol showed significantly lower weight compared to the arthritis alone and control groups (*P* < 0.05) (Figure 2).

### 3.4. Histopathology

**Figure 3:**
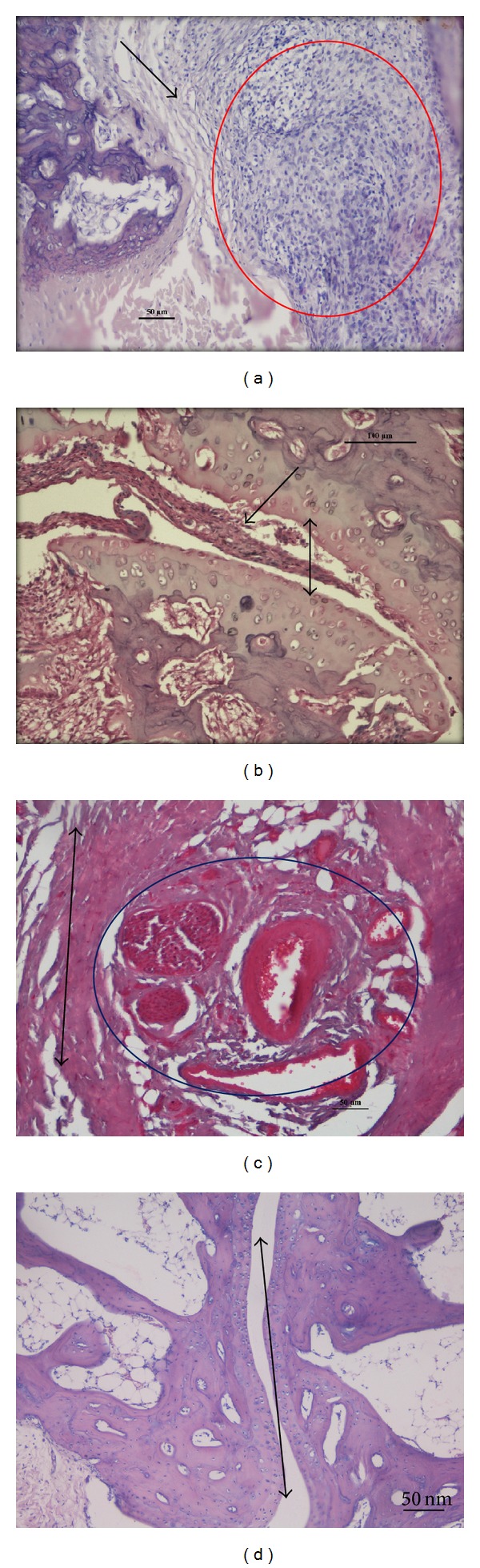
Histopathological analysis of joint morphology (H&E-200x). (a) Photomicrograph of arthritis group, showing synovial fibrosis (black arrow) and inflammatory infiltrate forming a pannus (red circle), (b) photomicrograph of arthritis + delta-tocotrienol, showing a significant reduction in synovial hyperplasia and inflammation (black arrow) and healthy joint space (black double arrow head), (c) photomicrograph of arthritis with glucosamine group, showing angiogenesis in the form of proliferating blood vessels (blue circle) and surrounding synovial healing scar tissue (black double arrow head), and (d) photomicrograph of control group, showing healthy normal joint space (double arrow head) with no inflammation or hyperplasia.

Histological sections were examined by light microscopy after H&E staining. All rats in the various arthritic groups showed significant changes in joint structure with varying degrees of arthritis changes. Changes observed included inflammation, cellular inflammation, joint space narrowing, synovial hyperplasia, erosion, and fibrosis. The joints of normal animals showed normal architecture with no swelling of the joint space. There was adequate gap and the articulating surfaces were lined by a healthy lining of cartilage, beneath which was the bony trabeculae. The synovium of these rats appeared healthy and there was no evidence of oedema or inflammation (Figure 3(a)).

Maximum degenerative changes were observed in the arthritis alone rats, where a feature of early and late-stage inflammation such as widening of joint space during early stages of the disease was observed. Severe congestion surrounding the joint space had resulted in oedema, dilation of blood vessels, and narrowing of the joint space later on. The surface surrounding the joint space showed erosion and degeneration (Figure 3(b)). Extensive synovial hyperplasia was also noted with increased cellular infiltration composed primarily of inflammatory cells (lymphocytes and plasma cells). Areas of granulomatous inflammation known as pannus formed in several areas with increasing fibrosis. The tocotrienol supplemented rats showed less severe changes when compared to arthritis. Inflammation was scarce with a marked reduction in edema and congestion. Only scatters of inflammatory cells were observed, suggesting that only moderate inflammation was present. There were few focal areas of fibrosis present, indicating healing of the joint. Vascular dilation was still present accompanied by moderate synovial hyperplasia. Areas of active inflammation and healing were also noted (Figure 3(c)). The joints of the rats in the glucosamine-treated group also showed a significant reduction in swelling. Microscopically, the orientation of joint space was predominantly healthy and morphological changes were minimal. There were focal areas of mild edema and scattered inflammatory cells, amidst healthy synovial tissue. The subsynovial regions showed good vasculature and areas of fibrosis, signifying that the process of healthy healing was taking place (Figure 3(d)).

### 3.5. Collagen-Induced Proliferation of Splenocytes

**Figure 4:**
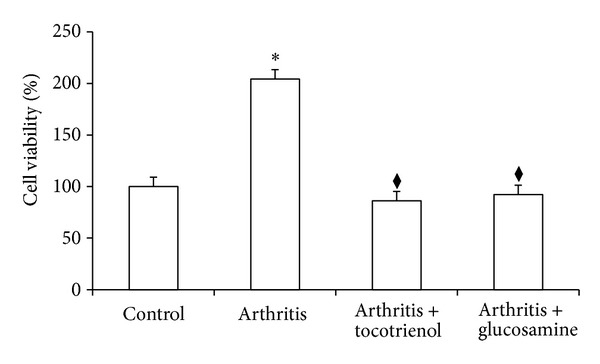
Proliferation of collagen-stimulated splenocytes from arthritic rats supplemented with delta-tocotrienol and glucosamine. Data are expressed as mean ± standard error. *P < 0.05: arthritis versus other groups; ^◆^ *P* < 0.05: arthritis with tocotrienol versus arthritis with glucosamine groups.

Splenocytes from the arthritic rats showed maximum cell viability when these cells were cocultured with 5 *μ*g/mL collagen for 24, 48, and 72 hours. At 72 hours, cell viability was at its peak with 85% compared with 59% for 24 hours and 62% for 48 hours. As the concentration of collagen increased, however, it was found that the viability of cells decreased to as low as 9%. Once the optimum concentration of collagen and incubation time were determined, this data (not shown) was used to determine the proliferation of splenocytes from the control and experimental rats. Proliferation of collagen-stimulated lymphocytes was quantified using the MTT assay. The results showed that the proliferation of collagen-stimulated splenocytes was reduced in animals that were supplemented with glucosamine and *δ*-tocotrienol (*P* < 0.05) (Figure 4).

### 3.6. C-Reactive Protein (CRP)

**Figure 5:**
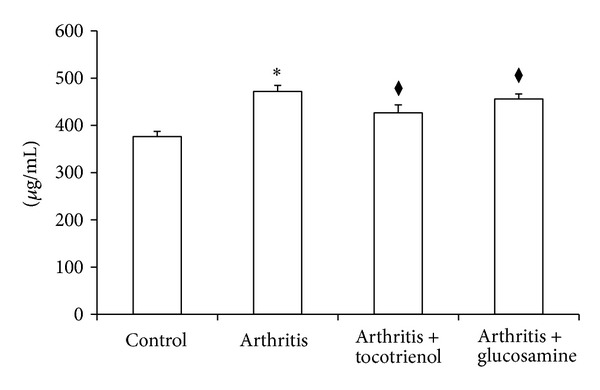
Effect of delta-tocotrienol on C-reactive protein concentration. Data are expressed as mean ± standard error of six rats per group. *P < 0.05: arthritis versus other groups; ^◆^ *P* < 0.05: arthritis with tocotrienol versus arthritis with glucosamine groups.

Plasma levels of C-reactive protein (CRP) were determined by using a commercial ELISA kit using the protocol recommended by the manufacturer (Millipore, USA). The arthritic group showed significantly (*P* < 0.05) elevated levels of CRP compared to the control group. There was a significant decrease in the CRP in the *δ*-tocotrienol and glucosamine-treated group (*P* > 0.05) when compared to the arthritis alone group (Figure 5).

## 4. Discussion

This study aimed to assess the therapeutic efficacies of *δ*-tocotrienol. In this study, several parameters which enable the elucidation of these potential benefits were investigated for these purposes.

Six joints of the hind-paws were assessed for changes in paw oedema. Only the hind-paws were chosen as they were the most significantly affected and literature has shown that this is a feature in collagen-induced arthritis [11]. In four out of the six joints assessed, tocotrienol had the most significant reduction in paw oedema when compared to all other groups. It is clear that tocotrienol treatment had a considerable impact on reducing paw edema.

Body weight of rats in all groups showed a linear increase with no unusual changes. There were no significant differences noted between the experimental groups. This was an important marker in showing that the animals were not in distress during the experimental period. There was a significant increase in the body weight in arthritis group after day 40 when compared with the other three groups. This increased weight could be due to water retention caused by edema in these animals. Most studies on CIA show rapid weight loss in rats during the period after induction of arthritis, which gradually increased with remission of the disease [12]. This was however not seen in this study.

Collagen-induced arthritis established in DA rats was a reliable model, with a 100% incidence of arthritis. There was consistent development of full-blown arthritis in at least one of the hind-paws with the distal interphalangeal joint being involved. No rats were severely disabled to warrant early sacrifice. Arthritis developed acutely in rats, with joint changes occurring much more rapidly than in human RA. This allowed for a more detailed observation of joint changes before and after treatment. The CIA is associated with unwanted features such as variable incidence, severity, and intergroup inconsistency [13] which were controlled by maintaining appropriate environmental conditions.

Macroscopically, all rats induced with arthritis showed similar signs and arthritis had peaked by day 25. Classical signs of CIA were observed, including symmetrical joint involvement typically involving the hind-paws, swelling, and erythema over the joints [14–16]. By the end of treatment period (day 50), treated groups showed an amelioration in signs and improved mobility of the joints. Histopathological changes correlated with macroscopic observations, including changes in paw oedema. Hallmarks of the CIA were noted and were present in varying degrees amongst the different groups. Untreated (arthritis only) rats showed maximum degenerative changes. Suppression of disease activity was seen in treated groups with the greatest changes in the *δ*-tocotrienol group.

Previous studies using animal models of arthritis showed that anti-inflammatory effects were attained through the inhibition of inflammatory mediators [17]. Tocotrienols have been described to inhibit these mediators, primarily TNF-*α* and IL-1*β* [17–20], which could possibly correlate with the attenuation histopathological changes found in these groups. The antioxidant qualities of tocotrienols are also a key in modulating joint injury by preventing free radical induced damage. Tocotrienols are known to possess a higher free radical quenching ability [21, 22]. Suppression of disease activity with *δ*-tocotrienols could be due to the fact that *δ*-tocotrienol is known to lower TNF-*α*, IL-1*β*, and nitric oxide levels [18, 19]. Glucosamine is a known potent antioxidant and has been used in osteoarthritis because of its ability to reduce joint damage [23]. This is consistent with our findings in which glucosamine exhibited disease attenuation property in arthritis rats.

Splenocyte proliferation was performed to determine whether treatment with *δ*-tocotrienol is associated with a protection against cell-mediated immunity. Proliferation of collagen-specific T-cells (CII-T) was assessed by the MTT assay. Firstly, conditions in which proliferation was maximal had to be established to determine accurate quantification of these cells. Therefore, optimisation on the concentration of collagen needed to stimulate appropriate amounts of CII-T was carried out in this study. Although the procedure for T-cell assay is well established, there seemed to be conflicting information in the literature as to the optimum concentration of collagen [24–27]. The results showed that, at a concentration of 5 *μ*g/mL with an incubation time of 72 hours, proliferation was at its maximum. Collagen toxicity towards cells increased at concentrations greater than this. This was the case for both the normal and the arthritic rats. It has been established that T-cell infiltration is directly correlated with the severity of arthritis [26]. The assumption that increased amounts of T-cells in the synovium occur as a result of clonal expansion to specific antigens has been explored previously [28]. In the case of CIA, the autoantigen is known to be collagen type II [26]. Therefore, it is safe to assume that high levels of CII-T cells indicate an increased disease severity. It has been reported that when introduced into the dermis, collagen type II is immediately captured by antigen presenting cells (APCs). This results in the activation and expansion of CII-T cells, initiating joint damage [5]. Therefore, by quantifying levels of CII-T cells, therapeutic benefits of *δ*-tocotrienol and glucosamine were assessed and compared. Using the set conditions, it was found that both *δ*-tocotrienol and glucosamine exhibited the significant suppression of CII-T cells when compared to the untreated groups. Both of these exhibited values close to those of the normal nonarthritic rat. In comparison with each other, *δ*-tocotrienol exhibited a higher suppressive power than glucosamine. As such, our observation that *δ*-tocotrienol reduced CII-T-cell proliferation may indicate a mode of protection offered by this isomer of vitamin E against inflammatory arthritis.

It is unclear however by which exact mechanism *δ*-tocotrienol was able to suppress the clonal expansion of CII-T cells. We hypothesise that it could be through one of two mechanisms: (i) direct suppression of the CII-T cells or (ii) upregulation of T-regulatory (Treg) cells. Direct suppression could arise from blocking the interaction between T-cells and APCs or prevention of T-cell infiltration into the synovium [28]. Treg cells have been proposed over recent years to inhibit the initiation or downregulate immune reactions in inflammation [29]. Studies have shown Treg cells to prevent proliferation and cytokine production of antigenic T-cells, thereby controlling inflammatory responses [28]. To determine which of these constitutes the underlying mechanism, further study needs to be done. A limitation of these findings is that it does not demonstrate a suppression of CII-T cells over time due to tocotrienol treatment. Thus, significant conclusions cannot be made that the tocotrienols offer protection against RA by reducing the number of T cells.

Biomarkers of inflammation have proven to be useful in the evaluation of disease progression and response to therapeutic intervention in a number of systemic inflammatory disorders, including RA. One such marker is C-reactive protein (CRP). An acute phase protein, CRP, is produced in the liver under conditions of systemic inflammation. It is reported to be a very useful marker of inflammation as its half life does not alter in health and disease states and it directly correlates with the intensity of pathological processes [12]. In clinically active human rheumatoid arthritis, levels of CRP are found to be increased [30]. This is translated across to animal models of rheumatoid arthritis where a similar process is observed [12]. One study demonstrated that high CRP levels are associated with incidence of total joint replacement in patients with arthritis and lower levels of CRP correspond to sustained suppression of the disease [12]. CRP levels have also been shown to be good markers for inflammation, bone degradation, and clinical well-being of patients with rheumatoid arthritis [12]. Studies have also shown that plasma CRP does not tend to rise substantially in response to inflammation in rats [31]. Plasma CRP level decreased significantly with *δ*-tocotrienol and glucosamine treatment in this study. The *δ*-tocotrienol and glucosamine groups still had significantly lower levels of CRP by the end of the experimental cycle compared to arthritis alone group [12, 19, 30]. Production in CRP is activated by synovial macrophages and fibroblasts mostly via a number of inflammatory cytokines including TNF-*α*, IL-1, and especially IL-6 [32]. These cytokines are similarly produced in abundance in RA; thus it can be said that lowered levels of CRP with tocotrienol treatment signify decreased cytokine production and consequently decreased disease activity.

## 5. Conclusions

In conclusion, this study has demonstrated that oral supplementation of *δ*-tocotrienol potently attenuates the development of progressive joint destruction in rats with CIA. This effect is due, in part, to their ability to inhibit T-cell proliferation, reverse histopathological changes, and inhibit production of proinflammatory cytokines. The properties exhibited by *δ*-tocotrienol showed promising outcomes against collagen-induced arthritis in this study. Therefore, there is clear evidence to suggest the potential benefits for this tocotrienol to be used as therapeutic agent in rheumatoid arthritis. Furthermore, insight into the possible mechanisms of this drug and disease should be uncovered to unleash a whole new realm of therapeutic possibilities.

## Figures and Tables

**Table 1:** Effect of delta-tocotrienol on percentage changes in paw edema in arthritis rats.

Days	Left hind limb joint			Right hind limb joint		
	Arthritis	Arthritis + tocotrienol	Arthritis + glucosamine	Arthritis	Arthritis + tocotrienol	Arthritis + glucosamine
Day 25	100 ± 2.2	100 ± 1.2	100 ± 1.5	100 ± 1.3	100 ± 2.4	100 ± 2.5
Day 30	98.37 ± 3.4	94.74 ± 2.8∗	92.35 ± 1.2	94.87 ± 4.1	92.05 ± 3.7	94.76 ± 3.2
Day 35	95.93 ± 3.7	88.05 ± 3.8∗	90.91 ± 4.1∗	92.71 ± 2.1	89.94 ± 2.8∗	91.45 ± 2.4
Day 40	92.05 ± 4.8	84.35 ± 5.4∗	88.75 ± 3.4∗	90.19 ± 3.7	85.45 ± 2.9∗	88.91 ± 1.1∗
Day 45	88.42 ± 4.9	80.75 ± 4.3^∗*◆*^	87.41 ± 2.8	88.99 ± 3.9	82.41 ± 4.1^∗*◆*^	84.65 ± 2.4∗
Day 50	87.24 ± 2.8	75.35 ± 2.7^∗*◆*^	82.15 ± 3.8∗	87.31 ± 2.8	78.94 ± 2.1^∗*◆*^	79.43 ± 2.8∗

Data are expressed as mean ± standard error of six rats per group. ∗*P* < 0.05: arthritis versus other groups; ^*◆*^ *P* < 0.05: arthritis with tocotrienol versus arthritis with glucosamine groups.
